# The experience of hospitalization in people with advanced chronic obstructive pulmonary disease: A qualitative, phenomenological study

**DOI:** 10.1177/17423953211073580

**Published:** 2022-02-04

**Authors:** Barathi Bakthavatsalu, Catherine Walshe, Jane Simpson

**Affiliations:** 1 Division of Health Research, 151268 Faculty of Health and Medicine, Lancaster University, Lancaster, UK; 2 International Observatory for End-of-Life Care, Division of Health Research, 151268 Faculty of Health and Medicine, Lancaster University, Lancaster, UK

**Keywords:** advanced COPD, repeated hospitalization, acute breathlessness, phenomenology, qualitative study

## Abstract

**Objectives:**

People with advanced chronic obstructive pulmonary disease (COPD) are frequently hospitalized, reporting high physical, psychological and spiritual suffering. Existing research focused on discrete aspects of hospitalization, such as care or treatment, yet lacks a complete picture of the phenomenon. The aim of this study is to understand the lived experience of hospitalization in people with advanced COPD.

**Methods:**

A qualitative, descriptive phenomenological approach was employed to study the phenomenon of hospitalization for people with advanced COPD. Unstructured interviews were conducted during hospitalization at a tertiary care hospital in India, in 2017, audio-recorded, and then transcribed. Giorgi's descriptive phenomenological analysis method guided the analysis.

**Results:**

Fifteen people with advanced COPD participated. Emergency admissions were common because of acute breathlessness, leading to repeated hospitalizations. Hospitalization gave a sense of safety but, despite this, people preferred to avoid hospitalization. Care influenced trust in hospitalization and both shaped the experience of hospitalization. Multi-dimensional suffering was central to the experience and was described across physical, psychological and spiritual domains.

**Discussion:**

Hospitalization was identified largely as a negative experience due to the perception of continued suffering. Integrating palliative care into the routine care of people with advanced COPD may enable improvements in care.

## Introduction

Hospitalization is common for those with advanced chronic obstructive airways disease (COPD).^1^ Admission is often related to exacerbations of breathlessness, frequently recurrent, as 27% of exacerbations are followed by a second event within 8 weeks.^2^ Readmission to hospital is therefore a regular pattern for people with advanced COPD, with 90 day readmission rates ranging from 16–48%.^3^ Hospital episodes are not only frequent, but also extended, as the mean length of hospital stay can be around 9 days.^4–6^ People experience being hospitalized in different ways, but generally, while people appreciate treatment, they can also associate hospitalization with uncertainty, distress and loss of control.^7–9^ Also people have a high symptom burden that affects the physical, psychological, social and spiritual aspects of individuals during hospitalization.^10^ Prolonged symptom burden causes high healthcare utilization and contributes considerable financial burden.^11^ It is critically important that there is a full understanding of the experience of hospitalization to facilitate high quality, holistic care.

A qualitative research approach can facilitate an understanding of individual experiences such as hospitalization. Qualitative studies have examined hospitalization in people with mixed stages of COPD, mainly from symptom burden, breathlessness, anxiety, and treatment perspectives.^12–15^ These studies provide some insight into the experience of hospitalization, however, not in the context of advanced COPD. Currently, there is more evidence from this perspective of the lived experience of people with advanced COPD in community settings, and their care needs.^16^ Studies on the experience of hospitalization in people with advanced COPD has mainly focused on elements of care and treatment.^17^ Therefore, it is important to study the experience of hospitalization from admission to discharge, to enable an understanding of the complete picture of the phenomenon.

A phenomenological approach, in particular, offers a rich account of the phenomenon being studied, such as the individual experience, to expound and present the essence of the phenomenon.^18^ Studies have adopted phenomenology as an approach to find the essence of a particular experience, such as the acute care experience, which indicates that this could be a valuable approach to study the experience of hospitalization.^9^ A phenomenological approach presents the phenomenon as it appears in its context, which can facilitate studying the experiences while preserving the individual culture and context.^19^ Current studies of hospitalization in advanced COPD are conducted in the Western context, which could have influenced the presentation of the phenomenon.^17^ Asian countries are not well-represented in previous studies on hospitalization; therefore, utilizing a phenomenological approach could be valuable in studying hospitalization in the Indian context. This study aims to explore the phenomenon of hospitalization in people with advanced COPD in India. Additionally, in the light of a lack of evidence focusing on the whole experience of hospitalization, the research question asked was: ‘what is the lived experience of hospitalization in people with advanced COPD?’

## Methods

### Study design

Qualitative, descriptive phenomenology following Giorgi's method was employed, as it provides in-depth exploration of the phenomenon.^20^ This study was reported following COREQ guidelines.^21^

### Philosophical underpinning

The epistemological underpinnings of descriptive phenomenology emphasizes consciousness of intentionality and phenomenological reduction or bracketing, which enable the study of a phenomenon.^20,22^ In this research, intentionally directing the researchers’ consciousness to the phenomenon as described by the participants facilitated studying the experience of hospitalization, as it appears in the context. Bracketing the researcher's assumptions related to hospitalization helped approach the phenomenon fresh to present the essence of the phenomenon.

### Population

Adults over the age of eighteen years with advanced COPD according to the Global Initiative for Chronic Obstructive Lung Disease (GOLD) stage III and IV^23^ and /or with clinical staging, and those who were currently hospitalized were the population of interest (Table 1).

**Table 1.** Inclusion and exclusion criteria.

Inclusion criteria	Exclusion criteria
COPD patients with GOLD stage III and IV;^23^ clinical symptoms such as worsening breathlessness, acute exacerbations with frequent hospitalization, use of a non-invasive ventilator and/or long term oxygen-dependency were also considered to identify the advanced stage. Adult patients > 18 years of age. Admitted to the pulmonary and geriatric medicine wards for a minimum of 24 h. Able to communicate fluently in Tamil and/or English.	Acutely ill participants who were unable to communicate and with cognitive impairment.

### Setting

This study was conducted in a tertiary care hospital in south India, which caters to the wider population of the city, as well as for people from the neighboring states. This is a private, Christian, religious hospital, which provides care for people from various religious and socio-economic backgrounds.

### Sample

A purposive, homogenous sampling method was employed, as this helped identify the potential participants who shared similar characteristics to study the experience.^24^ According to Giorgi's phenomenological method the adequacy of sample size is determined by saturation of themes.^25^ Data collection was stopped when themes were saturated.

### Recruitment

Potential participants were identified from the admission records of pulmonary and geriatric medicine wards by the hospital staff. Participants indicated their willingness to participate to either staff or the first author (BB). Then, BB took written consent from willing participants, after explaining the voluntary nature of participation and potential to withdraw from the study. Fifteen participants were approached and all agreed to participate in the study.

## Data collection

To explore the phenomenon in depth, descriptive and structural questions were used.^25–27^ Descriptive questions were used to understand the overall experience of hospitalization, and structural questions to clarify the phenomenon of hospitalization. Individual, face-to-face, unstructured interviews were conducted in the Tamil language, employing open-ended questions (appendix 1). All the interviews were conducted at the hospital either at the bedside or in the adjacent counselling room by BB. Participants’ family members were allowed to be present during the interview but it was explained that they could not participate in the interview, given the idiographic focus on the person with COPD. Interviews were recorded with one or two temporary pauses of a few minutes, allowing time to manage any breathlessness or cough. In case of emotional upset, the first author BB provided initial support by active listening, and visited them next day in the ward to find out whether further psychological support was required. None of the participants requested this further psychological support.

Interviews were audio-recorded on an encrypted digital voice recorder then, transferred to a password protected computer. Data collection and coding were done simultaneously, so any new topics found from the analysis could be added to the next interview. The author's (BB) previous knowledge about advanced COPD including textual and experiential knowledge was bracketed to avoid subjectivity, in order to get a fresh view of the phenomenon.^28^ The process of bracketing was followed from the time of data collection until data analysis.

## Data analysis

BB transcribed all the audio recordings verbatim into the Tamil language and then, translated these into English. They were back-translated into Tamil by a bilingual expert to check the accuracy of the translation. Employing back-translation ensured that the meaning of the translated text in English stayed close to the original language; back-translation not only reduces translation errors, but also ensures the validity of translation.^29,30^ Any challenges in translation were discussed with the translator and resolved before the final version of the transcript was prepared and this final, verified transcript was used for analysis.

Data analysis followed Giorgi's five step phenomenological analysis method.^20^ Transcriptions were imported into the NVivo 11 software to enable data management and analysis. Data were coded by BB and checked by CW, for the consistency of coding. Firstly, the text was read to understand its wholeness. ‘Bracketing’ was used to suspend presuppositions by writing down (BB's) thoughts related to hospitalization and COPD.^31^ Then, the text was re-read to capture the ‘meaning units’, which are parts of the text relevant to hospitalization. Thirdly, meaning units were segregated and coded. The fourth step was to transform the meaning units into a scientific expression, which involved changing the participants’ description from the first person to the third person expression, and to a formal, scientific language, in order to integrate a similar description across all the transcripts. Finally, themes were developed from the transformed meaning units; ‘eidetic reduction’ was employed to include only the essential elements of the phenomenon by eliminating the non-essentials and repetitions. Identifying redundant elements through this process determined data saturation.^20^ The description of the phenomenon of hospitalization was written from the transformed meaning units of the main themes.

### Trustworthiness

Trustworthiness in qualitative studies refers to transferability, credibility, dependability and confirmability.^32,33^ Transferability is determined by giving a thick and rigorous description of the findings of the phenomenon of hospitalization which enables the transferability of the findings to a similar context.^32^ Credibility refers to the researcher's ability to produce an accurate data which is facilitated by employing ‘eidetic reduction’ to eliminate the irrelevant constituents; also the sufficiency of description, which is indicated by absence of repetitions in the description of the findings, indicated credibility.^20^ Dependability relates to the replicability of findings, which is facilitated by meticulously employing the steps of Giorgi's phenomenological analysis for analysing the data.^20^ Confirmability refers to the measures taken to ensure neutrality. Employing bracketing to avoid the researcher's previous experiences and assumptions from interpreting the data facilitated neutrality. Writing down and updating the researcher's thoughts related to the phenomenon and consciously avoiding these thoughts during data analysis helped practise the process of bracketing.

### Study team

This study was undertaken as part of a completed PhD by the first author (BB). The other authors are researchers with PhDs, with extensive experience in research in palliative care and psychology, respectively. The first author had earlier worked as a palliative care physician in the research setting; however, participants were not aware about her previous employment in the hospital and no participants had received clinical care from her.

### Ethics

Ethics approval was obtained from the authors’ host institution, Lancaster University Ethics Committee, (ID: FHMREC17006), Institutional Ethics Committee, St. John’s Medical College, India (ID: IEC/169/2016), and from the Indian Council for Medical Research (ID: 5/8/4-31).

## Findings

Fifteen participants were interviewed from August to December 2017. Five women and ten men were interviewed and their ages ranged from 61–83 years (see Table 2). The mean interview time was 26.06 min with a range of 20.2–30.5 min.

**Table 2.** Demographic characteristics.

Baseline characteristics	
Gender (n)	
Female	5
Male	10
Age	61–83 (years) Mean 66.2
Religion (n)	
Hindu	11
Christian	4
Time since diagnosis of COPD (years)	7–15 (range)
Oxygen therapy (n)	
Non-invasive ventilation	4
Nasal oxygen	1
Intermittent oxygen	10
Co-morbidities (n)	
Hypertension	7
Diabetes	3
Heart disease	2
Dyslipidemia	10
Number of days admitted	2–18 Mean 4.73
Hospitalization in past one year	1–5 Mean 2.26
Route of admission (n)	
Emergency department	10
Outpatient department	5

Four key constituents were developed: repeated hospitalization, perception of care, trust in hospitalization and multi-dimensional suffering. The essence of the phenomenon is presented in the following sections. The sub-constituents are organized under each main constituent.

## Repeated hospitalization

### Emergency admissions

Emergency admissions were common in the advanced stage of COPD due to the immediate treatment required for acute breathlessness. Although quick attention and immediate relief of breathlessness was appreciated, delays were reported both in deciding admissions and transfer to the ward. The delays were either due to the unavailability of a bed or a long queue of other patients waiting to be admitted:

“*I came morning 9am to the emergency department, evening 8pm only I got a bed.”* (P8)

At times, emergency department physicians were not aware of participants’ medical history, which caused confusion in treatment decisions:

“*No. Nobody explained…my son called up and asked the doctor to know why I needed ICU treatment.”* (P13).

Emergency admissions often involved quick medical decisions and immediate treatment, which, at times, could be overwhelming and anxiety provoking.

### Familiarity and reluctance towards hospitalization

A sense of familiarity with the treating staff and the routine procedures of hospital were perceived, during the course of repeated hospitalization. Familiarity with staff created a sense of safety and control with the belief that their problem would managed, which reduced anxiety:

“*They [doctors] give good treatment during emergencies…now I get the confidence that I will survive for some more days.”* (P6)

Feeling safe and being cared for helped the development of trust with staff. Having gone through the routine hospital procedures did create a sense of familiarity but, at times, complying with hospital routines was also perceived as troublesome despite an acceptance of their necessity:

“*They take blood tests and wake me up at 4 am in the morning; again some other staff comes and asks for tests. But they have to do their duty, I can't complain about it.”* (P3).

But the noisy and dirty hospital environment caused a negative impression about hospitalization. Particularly, noise from neighboring patients and medical equipments caused sleep disturbance during hospitalization:

“*When neighbour patients snore like tearing the whole building apart; I try to tie a towel around my head; I even thought of plugging my ears with cotton…”* (P12)

Apart from this, a sense of being a burden was perceived as the family provided care, spending both their money and time. Repeated hospitalization was perceived to cause an inability to fulfil family responsibilities, such as being able to cook for the family and look after grandchildren. However, participants seemed to understand the necessity of hospitalization and, therefore, accepted its inevitability:

“*What can I say…shall I remain at the hospital thinking that I will be fine if I go to hospital or shall I remain at home thinking that I would become alright at home?”* (P11)

Familiarity and a sense of safety created a positive impact on hospitalization but an unclean hospital environment and care burdens caused a negative impression.

## Perception of care

### Rewards and burdens of treatment

Immediate medical attention was perceived to help restore normal breathing and relieved the fear of imminent death. Both timely treatment and the expertise of the staff were considered to be components of efficient treatment, which enabled independence to be regained in a short time and then, a return to usual life:

“*…they [staff] give immediate treatment… just two days are sufficient for them… they change me to a normal man…”* (P15)

This also facilitated developing trust with staff, as well as trust in this particular hospital. While treatment brought immediate relief, participants were aware that the effect of treatment would not last for a long time, and understood the need for repeated treatment due to the nature of the illness:

“*I am OK now…* *within two days I will go home; but it may come again. I know that also.”* (P3)

Particularly, intensive care unit treatment was perceived as difficult; having tubes inserted all around the body and being hooked on to the oxygen machine created a sense of isolation. On occasions, not being aware of the reason for the intensive care unit admission was overwhelming:

“*No… nothing they [staff] said… just for nebulization, why should I be in the ICU?”* (P15).

While treatment was regarded as beneficial, at the same time, it was also perceived as burdensome.

### The attitude of the staff

The caring attitude of staff providing immediate attention, dedicated care by taking account of every small detail, listening and a good explanation of the treatment plan facilitated both a good relationship and development of trust with staff. Participants felt that staff provided good care, despite being busy and did everything to restore their health to normal:

“*The way the doctors treat us and the quick response, that itself makes me feel happy.”* (P3)

The caring attitude of staff encouraged individuals both to engage actively in the treatment and develop trust with them.

On some occasions, an unpleasant attitude from a member of staff, such as not paying attention, ignoring emotional concerns and failing to explain treatment plans caused emotional upset to the participants. Being direct and abrupt was felt unpleasant when communicating the prognosis. On occasions, doctors did not seem to be concerned about the presence of others and did not ask for the consent of the participant before discussing the prognosis more publicly:

“*A doctor told me that 98% of your lungs are dead [laughs]; my wife cried. He [doctor] is saying right to my face while my wife was with me.”* (P8)

Being insensitive to participants’ emotions and failing to discuss future treatment plans were perceived as being indicative of an uncaring attitude.

Most individuals did not completely understand their prognosis. However, individuals felt that the doctors should know when to initiate the conversations about prognosis, and to explain the treatment plan:

“*I don't ask usually; they [doctors] are here to tell me, if there is anything that I should know.*” (P9)

Insufficient information about prognosis and future treatment plan often caused a perception of inadequate care during hospitalization.

## Trust in hospitalization

Immediate symptom relief improved independence and participants’ ability to carry out daily activities during hospitalization, which established trust in hospitalization for continued care. Decisions about choosing a hospital were informed by good impressions from past experiences and the opinion of family and others. Individuals’ previous, good experience in this particular hospital shaped their trust in hospitalization:

“*Here, they make sure that the patients get relief. I came to this hospital because I had hope in them.”* (P6)

Further, an unreasonably high healthcare cost and poor quality of treatment was reported in other private hospitals, which reduced trust in hospitalization. Facilities available in the hospital, and expertise of the staff also influenced the decision of the choice of hospital:

“*No.it is neatly done here…all equipments are available and drawing blood is pretty smooth.”* (P8)

The opinion of family members and friends also played a role in choosing this particular hospital. The belief that this hospital held the value of Christian religion, which suggested good care and a high chance of getting well, seemed to influence the decision:

“*…that belief that this is a Christian hospital. Also my mother-in-law has confidence in this hospital.”* (P12)

Both individuals’ experience and their family's opinion about the hospital, which relates to the perceived moral and religious values of the hospital, seemed to be important in developing trust in hospitalization.

## Multi-dimensional suffering

Suffering was reported as multi-dimensional and affected the physical, psychological and spiritual aspects of the individuals.

The experience of breathlessness was described in many different ways: a choking sensation in the throat, a pulling sensation in the chest, a sensation of heaviness and/or tightness, perception of a rock-like chest or extreme difficulty in inhaling air. For some it was an intense struggle just to take a breath:

“*I feel as if it is blocked [breath]; air is not getting inside; it keeps coming outside.”* (P7)

The unpredictable nature of breathlessness and its severity devastated participants’ daily life. Getting breathlessness in the toilet or in similar situations seemed to impact their dignity:

“*… it [breathlessness] comes suddenly. What am I supposed to do then? As if I am going to pee or poop now [without control].”* (P1)

Fatigue accompanied with breathlessness restricted participants’ ability to do simple daily tasks, such as eating, which caused dependency on others*.* Feelings of low self-esteem and being a burden to others due to dependency were at times overwhelming:

*“Why am I like this? How long will my children take care of me?”* (P2);

“*Now I know for sure that this [illness] will not leave my body…isn't it?”* (P13)

Fatigue with dependency both indicated incurability and contributed to high symptom burden which caused a negative impact on the experience of hospitalization.

### Psychological and spiritual distress

Anxiety was experienced during breathlessness due to varied reasons. Some thought that it was natural to get scared during breathlessness. Being alone in the hospital bed during breathlessness caused fear, which was relieved by someone's presence or immediate medical attention. However, participants felt that they could not express their fear to anybody during hospitalization, as they felt staff were primarily there to take care of physical symptoms. Sensation of imminent death during acute breathlessness caused considerable spiritual distress.

Perceiving life was purposeless created a sense of emptiness in life and caused death wishes during hospitalization. Some participants perceived that death was better than living with suffering every day, while others expressed that they were not afraid of dying:

“*…when the time comes, I will go; if my time is good, I will be alive for some more time.”* (P9)

Nevertheless, reflecting fulfilment in life encouraged hope for the future, and to find meaning in life. Being able to complete family duties, such as raising children, providing education and seeing children married were regarded as important duties in the Indian culture, which gave a sense of fulfilment in life and hope around the future:

“*…whether it becomes cured or not, I need to be alive now; I have to get my son married…”* (P2)

In general, religious belief helped people accept suffering and death as part of life and participants continued to trust God, despite suffering, to provide a peaceful death and eternal life. However, participants did not seem to have had an opportunity to discuss death and dying concerns with staff during hospitalization. The depth of multi-dimensional suffering indicated persistent suffering despite frequent hospitalization.

## Discussion

This is the first study to take a holistic approach to understanding the lived experience of hospitalization in people with advanced COPD. Phenomenological analysis yielded four key constituents: repeated hospitalization, perception of care, trust in hospitalization and multi-dimensional suffering. This study found that the essence of the phenomenon of hospitalization had both positive and negative aspects; however, the overall experience was negative due to persistent, multi-dimensional suffering.

Both positive and negative views of hospitalization could be held simultaneously, which depending on the expectations about hospitalization and healthcare delivery. Studies conducted in cancer and other chronic illnesses report that hospitalization is a continuum of positive and negative experience, which is influenced by the seriousness of illness, expectations from hospitalization and interpersonal relationships with staff.^34,35^ The experience of hospitalization can see a co-existence of positive and negative, confirming the balance between expectations versus healthcare delivery.^17^ This empirical study found that care and treatment and communication were expressed as both positive and negative, which indicates that the phenomenon of hospitalization could be complex and variable*.*

Persistent, multi-dimensional suffering included physical, psychological and spiritual aspects, which caused a negative impact on the experience of hospitalization. This present study found that breathlessness was relieved with treatment, but it kept recurring during the course of hospitalization, which caused continuing suffering. While breathlessness was given immediate attention, other psychological and spiritual issues were neglected. Participants in this study were hesitant to discuss their anxiety during hospitalization, as they thought that staff were trained to treat only physical symptoms, which led to prolonged distress. Studies report that persistent psychological distress after discharge could be one of the causes for readmission in people with advanced COPD, which also contributed to a negative experience of hospitalization.^36,37^ Death and dying concerns were the main spiritual issues reported that remained unaddressed during repeated hospitalization. Previous studies conducted in people with advanced COPD do not report spiritual concerns, despite their connection with symptom burden and impact on quality of life.^38,39^ Studies which have examined hospitalization in both acute and non-acute settings identified that high symptom burden is the main contributor for a negative perception of hospitalization; however, these studies do not take account of psychological and spiritual issues into the conceptualization of symptom burden.^34,35,40^ Continuing physical, psychological and spiritual suffering could have contributed to a largely negative perception of hospitalization in this study.

This empirical study also found that poor communication and staff attitudes caused a predominantly negative perception. Studies confirm that communication and staff attitude are the major reasons for negative experience of hospitalization in individuals with other chronic illnesses.^40–42^ Poor explanation of treatment and inadequate explanation of disease prognosis caused uncertainty around the future which, in turn, had a negative impact on the experience. Discussing prognosis is known to be particularly challenging in acute care settings and the unpredictable disease journey of COPD further complicates the identification of advanced stage.^43^ However, utilizing effective communication skills, avoiding confrontations and setting realistic short-term goals could help in communicating disease prognosis.^44,45^ Staff attitude is another major influencing factor for patient satisfaction with hospital care which also determines the experience of hospitalization.^46,47^ This empirical study showed that attitudes such as not paying attention and ignoring emotional concerns caused a negative perception. Unclear disease prognosis compounded by poor communication seemed to impact the experience more negatively.

### Conceptualization of hospitalization

**Figure 1.**
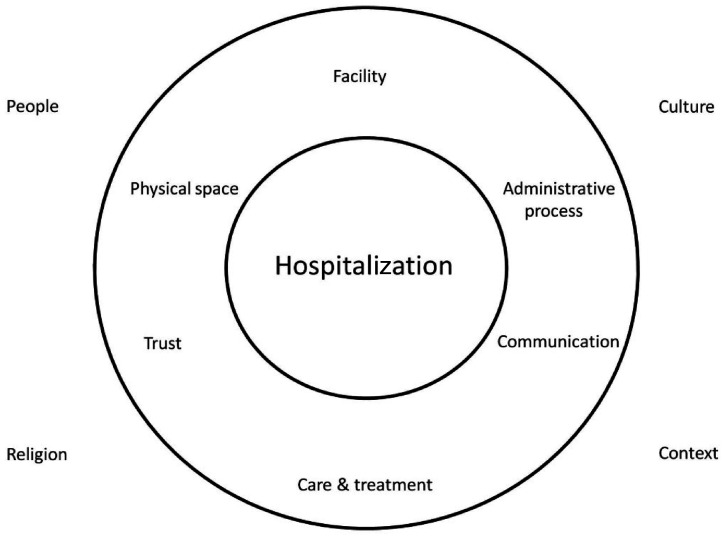
Conceptualization of hospitalization in advanced COPD.

Limited evidence exists on what constitutes the concept of hospitalization. Hospitalization has been studied both in varied chronic illness conditions and in various hospital settings, from acute care to ordinary inpatient care, but mainly from a care and treatment perspective.^42,48,49^ From this research, the conceptualization of hospitalization seems to include two main elements: firstly, the hospital as a physical place and a facility for the sick; secondly, being hospitalized which includes the multi-faceted aspects of administration, treatment and care, and communication. These two main elements are integral parts of each other but their sub-elements may be varied and together they contribute to the experience of hospitalization (Figure 1). This study identified trust in hospitalization as one of the contributing sub-elements along with other sub-elements. Care and communication, and trust seemed to have a contrasting perception of both positive and negative elements, which indicates the dynamic nature and complexity of these sub-elements.

The complexity of these elements mainly depend on the nature and depth of care required, the expectation of the individuals and the competency of the staff involved.^42,49^ This conceptualization of hospitalization should, therefore, be understood from the specific context of this research, such as varied socio-cultural and religious backgrounds of the participants, and from the perspective of repeated and often emergency hospitalizations for a chronic and advanced stage of illness (Figure 1)*.* These dimensions in themselves are not complete, as hospitalization is a broad, varied and complex phenomenon and could be conceptualized differently in other contexts and cultures.

### Strengths and limitations

This is the first qualitative study to capture the experience of hospitalization in people with advanced COPD. Previous studies on hospitalization have mostly been from a Western perspective, hence those findings may not be transferable to countries with a different socio-cultural context, such as India, which has a high prevalence rate of COPD and hospitalization.^50^ This is the first study on the experience of hospitalization in the Asian/Indian setting, where research on COPD care is scarce. This study included participants with varied socio-cultural backgrounds that enriched the experience of hospitalization.

Interviews were conducted in the hospital, to capture the phenomenon afresh, which helped overcome the challenge of relying on the memory of participants.^51^ Interviewing at the hospital facilitated the exploration of real-time experience capturing the time-space dynamics in hospitalization, which helped narrating all the nuanced aspects of the experience.^52^

### Limitations

The sample had more male participants than female, although it does reflect the prevalence of COPD in India.^50^ Nonetheless, this could mean that important aspects of the COPD experience which some research has shown might be more pertinent to women (such as loss of role as a home-maker, altered physical appearance, and a lack of confidence to cope with the illness alone), could also have influenced the experience.^53^ The relatively short interview length (*M* = 26 min) was likely due to the inherent challenges of interviewing participants with breathlessness and extreme physical weakness, but could have limited the depth of exploration of certain aspect of the experience.^54^ Finally, as participants were still hospitalized, the discharge aspect did not emerge during the interview.

## Recommendations for practice and policy

### Practice

Poor communication, especially during emergency admissions, has been identified as an area that requires improvement. Communication training for staff, specifically in discussing end-of-life care, could enable staff to be more effective in communicating sensitive topics.^55–58^ Emergency staff could also take support from palliative care professionals when required. Staff should be able to identify psychological distress and access help from a psychologist when required. Screening of spiritual concerns should be included in the routine care of people with advanced COPD to identify spiritual distress.

### Policy

The preference to discussing advance care planning needs to be addressed, as this might vary in other international settings that some people want less or more information. This aspect needs to be considered by the policy developers and clinicians to provide tailored information.^51,59^

## Future research

The feasibility of adopting an integrated care plan to ensure continuity of care, as patients often require a transfer between pulmonary and palliative care, and the benefits and challenges in implementing this plan, needs to be researched in the Indian context.^60^

This research captured the experience of hospitalization as a snapshot, which may not be adequate to understand the varying perception of hospitalization. Since hospitalization is a complex and dynamic experience, a longitudinal, qualitative study should be considered to understand the experience of repeated hospitalization over time.^61^

## Conclusion

This study found that the phenomenon of hospitalization included both positive and negative aspects, depending on the individuals’ context-specific needs and expectations*.* However, the overall experience of hospitalization was perceived predominantly as negative due to persistent, multi-dimensional suffering, despite repeated hospitalizations. This indicates a lack of a holistic approach to people hospitalized with advanced COPD. A palliative care approach could help providing a holistic care for individuals with multi-dimensional suffering during hospitalization.^10^ Early integration of palliative care into the routine care of COPD is required to improve the care of individuals with advanced COPD.

## Appendix

### Appendix 1- interview guide

The interview will use a broad, open question to begin the interview. It will use indicators to extract the information from the participant. Some of the indicators that might be used in the interview are stated below.

*Opening question*

Can you please tell me about your experience of being hospitalized?

*Indicators/Prompts*

1. Can you tell me more about it?
2. How does that make you feel?
3. Could you describe it in little more detail?
4. How exactly does that affect you?
5. What made you feel like that?
